# Case Report of Dual Variants in *SLC1A4* and *APOE* : A Possible Link Between Amino Acid and Lipid Metabolism

**DOI:** 10.1002/ccr3.73014

**Published:** 2026-06-21

**Authors:** Mohammed Qaisiya, Kenana Altell, Wasef Alhroub, Mahmoud Ramlawe, Elisabetta Battocchio, Fabrizia Guarnieri, Mu'taz Altamimi

**Affiliations:** ^1^ Faculty of Pharmacy, Department of Medical Laboratory Science Hebron University Hebron Palestine; ^2^ Faculty of Medicine, Pediatric Neurology Hebron University Hebron Palestine; ^3^ CNR Institute of Neuroscience Pisa Italy

**Keywords:** APOE, L‐serine, progressive microcephaly, SLC1A4, spastic tetraplegia, thin corpus callosum

## Abstract

Spastic tetraplegia, thin corpus callosum, and progressive microcephaly (SPATCCM) is a rare autosomal recessive neurodevelopmental disorder linked to biallelic variants in the *SLC1A4* gene, which encodes the ASCT1 amino acid transporter critical for neutral amino acid uptake in neurons. While *SLC1A4* mutations are well established in SPATCCM, the potential impact of additional genetic factors on disease progression remains unexplored. We report a rare case of an 8‐month‐old male with severe global developmental delay and progressive microcephaly, identified with whole‐exome sequencing to have a novel combination of homozygous *SLC1A4* (p.Arg457Trp) and heterozygous *APOE* (p.Cys130Arg) mutations. Neuroimaging revealed severe hypomyelination and diffuse white matter abnormalities. This is the first reported case of SPATCCM with an *APOE* variant, implicating a potential interplay between amino acid transport deficiencies and lipid metabolism in brain development.

## Key Clinical Message

This case highlights a rare dual‐variant scenario in Spastic Tetraplegia, Thin Corpus Callosum, and Progressive Microcephaly (SPATCCM), where a homozygous SLC1A4 mutation co‐occurs with a heterozygous APOE ε4 variant, suggesting that interactions between amino acid transport and lipid metabolism may exacerbate neurodevelopmental impairment.

## 1 Introduction

Spastic tetraplegia, thin corpus callosum, and progressive microcephaly (SPATCCM, OMIM #616657) is a rare autosomal recessive neurodevelopmental disorder characterized by severe developmental delay in early infancy, intellectual disability, inability to achieve independent walking or speech, and in some cases epileptic seizures. This condition was first reported in two siblings born to Ashkenazi Jewish consanguineous parents in which whole exome sequencing revealed a rare homozygous missense mutation in *SLC1A4* (c.766G>A, p.E256K) [1]. The E256K mutation was additionally reported in 9 patients from 7 unrelated families of Ashkenazi Jewish, and a different homozygous variant in *SLC1A4* (p.R457W) was identified in one girl born to consanguineous Palestinian parents [2]. Two additional Ashkenazi Jewish patients were reported with SPATCCM, one carrying the p.E256K (c.766G>A) homozygous missense mutation and the other with two compound heterozygous mutations (c.766G>A and c.945delTT) [3]. Other genetic biallelic variants have been identified in one patient from Ireland (nonsense mutation p.W453*) [4], one patient from Italy (p.G381R) [5], one patient from Turkey (p.G374R) [6], one Czech patient (p.R457Q) [7] and four affected children from Pakistan (two with homozygous nonsense mutation p.Y191* and two compound heterozygous for p.N324Tfs*29 and p.S181F) [8, 9]. A *de novo* heterozygous three amino acid duplication in *SLC1A4* (L86_M88dup) was recently reported as a dominant‐negative variant causing autosomal dominant disease [10].

The *SLC1A4* gene encodes for the sodium‐dependent neutral amino acid transporter ASCT1 (Alanine/Serine/Cysteine/Threonine and Glutamate Transporter 1) that has a fundamental role in neuronal growth, synaptic plasticity, sphingolipid synthesis and regulation of the intracellular ion gradients [11]. In particular, L‐serine plays a crucial role in neuronal development, neurotransmission regulation, cognitive function and remyelination [12]. L‐serine is synthesized from the glycolytic intermediate 3‐phosphoglycerate in three consecutive steps. This biosynthetic pathway is largely confined to astrocytes; the generated L‐serine is shuttled to neuronal cells through ASCT1 and converted to D‐serine, which acts as a neuromodulator [13]. The human mutations identified in *SLC1A4* have been found to cause ASCT1 *loss‐of‐function*. The two main ASCT1 missense mutations, p.R457W and p.E256K, lead to an abolished or markedly reduced L‐serine transport, respectively, without affecting the membrane localization of the protein [14]. Nonsense and frameshift mutations probably lead to the production of a truncated non‐functional ASCT1 or to nonsense‐mediated mRNA decay. The L86_M88dup mutation introduces a 3‐amino acid duplication in the protein, which causes defective N‐glycosylation and altered ASCT1 membrane localization thus reducing L‐serine transport [10].

The *APOE* gene encodes for the apolipoprotein E protein (APOE), known for its role in lipid metabolism and transport. The gene has three allelic isoforms, with the respective encoded proteins differing for two amino acid residues: ε2/APOE2 (Cys130 and Cys176), ε3/APOE3 (Cys130 and Arg176), and ε4/APOE4 (Arg130 and Arg176). The ε4 allele has been recognized as the main genetic risk factor for late‐onset Alzheimer's disease, and its expression has been linked to age‐related cognitive dysfunctions [15]. In addition, emerging evidence suggests a potential role of APOE in neurodevelopment. Indeed, children and young adults carrying the APOE4 allele exhibit altered brain morphology, with reduced hippocampal volume and cortical thickness, as well as impaired performance on attention and working memory tasks [16, 17, 18].

Here we describe an extremely rare case characterized by microcephaly, tetraplegia, and spasticity, in which we identified the homozygous p.R457W *SLC1A4* mutation in combination with the heterozygous p.C130R APOE4 variant.

## 2 Case Presentation/Examination

A 8‐month‐old male presented with concerns of microcephaly and global developmental delay from a Palestinian family with 3 siblings. The proband child was born full‐term through normal vaginal delivery without maternal and neonatal complications prenatally and postnatally, weighing 2.7 kg, with exclusive breastfeeding, complete routine vaccination schedule and no history of NICU admission up to his age. His parents were referred for developmental assessment due to a small head size. Physical examination revealed no dysmorphic features, a head circumference below the 5th percentile, body weight between the 3rd and 15th percentile, and length in the 10th percentile. The baby exhibited weak eye contact, good head support, normal visual behavior, and no long tract signs, but was unable to roll over or reach for objects or to move them from hand to hand. Further examination showed a well‐looking, hydrated baby with no dysmorphic features, no skin rash, normal ENT findings, and a normal chest, heart and skeletal exam. At 17‐months old, a gastrostomy tube was inserted to avoid dehydration and electrolyte imbalance because of reported dysphagia. His weight remained constant at 10 kg from 8‐months to 17‐months old, but over the previous period, he had experienced weight loss, dropping to 7 kg till 2‐years old due to severe dehydration and severe vomiting. Neurological assessment revealed exaggerated deep tendon reflexes in upper and lower limbs with no clonus, and a positive Babinski sign. At the age of 2 years, he started to have multifocal epileptiform activity and hypothermia (31°C–33°C). Laboratory investigations, including CBC, electrolyte levels and kidney function tests were within normal limits while AST (2870 U/L) and ALT (1969 U/L) were highly increased suggesting severe liver injury. Brain MRI findings showed diffuse white matter abnormalities and severe hypo‐myelination (Figure 1).

**FIGURE 1:**
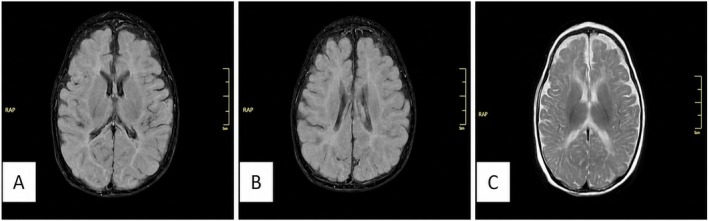
Axial MRI images of the brain from the case report (A, B): FLAIR sequences showing reduced white matter myelination, consistent with delayed myelination or hypomyelination. (C): T2‐weighted sequence displaying normal ventricular system configuration without midline shift or intracranial hemorrhage. The findings suggest underdevelopment of the corpus callosum and abnormal white matter myelination.

## 3 Methods

Whole‐exome sequencing (WES) was performed upon informed consent from the parents. DNA was extracted from dried blood spot and sequencing was performed on genomic DNA enriched for the exome using a sequence capture method. Direct sequencing of the amplified captured regions was performed using 2X150bp reads on Illumina next generation sequencing (NGS) systems. A base was considered to have sufficient coverage at 20X and an exon was considered fully covered if all coding bases plus three nucleotides of flanking sequence on either side were covered at 20X or more. Alignment to the human reference genome (GRCh37) was performed and annotated variants were identified in the targeted region. Primary data analysis was performed using Illumina bolfasta converter v2.19. Secondary analysis was performed using Illumina DRAGEN Bio‐IT Platform v.3.10.8. Tertiary data analysis was performed using SpEff v5.0 and PerkinElmer's internal ODIN v.1.01 software (PerkinElemer genomics, USA).

Two variants were identified: the first one is a previously reported homozygous pathogenic variant in the *SLC1A4* gene (c.1369C>T, p.R457W), associated with SPATCCM; the second one corresponds to the ε4 allelic variant of the *APOE* gene (c.388 T>C, p.C130R) (Table 1). Genetic counseling confirmed an autosomal recessive inheritance pattern, with reported parental consanguinity. No WES was performed for parents or brother and sister, but they were advised to seek genetic counseling if they plan any future pregnancy. Following the family history, many dead cases were reported with no investigated reasons (at the ages of 2‐years‐old to 7‐years‐old). Another 3‐year‐old male was diagnosed with SPATCCM and identified by WES at the age of 1‐year‐old, carrying the same mutation of SLC1A4 (but not the APOE variant) as the proband described in this case report (Figure 2). Despite the rarity of the SLC1A4 mutations worldwide, the pedigree of this family raises an important concern regarding the frequency of parental consanguinity cases within the regional population and the need for a better genetic counseling approach.

**TABLE 1:** Sequence variants in the case reported.

Classification	Gene	Exon	DNA change	Protein change	Zygosity	Inheritance	OMIM	Associated disease
Likely Pathogenic	*SLC1A4*	8	c.1369C > T	p.Arg457Trp	Homozygous	AR	600,229	Spastic tetraplegia, thin corpus callosum, and progressive microcephaly (SPATCCM)
Risk allele	*APOE*	4	c.388 T > C	p.Cys130Arg	Heterozygous		107,741	late‐onset Alzheimer's disease

**FIGURE 2:**
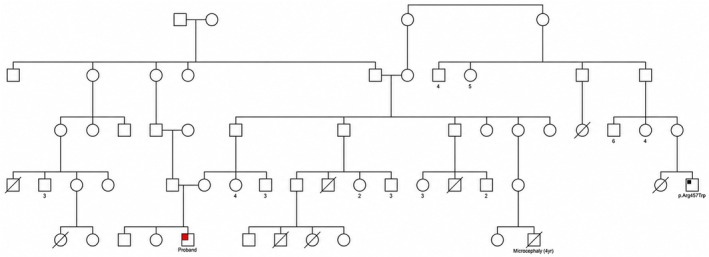
Pedigree of the SPATCCM case report with SLC1A4 mutation. The pattern of inheritance is autosomal recessive. Square donate men, circles women and the slashes are individuals who have died. The proband (red square) is the third child for parents in a family with consanguinity. The individual with the (black square) is a 3 years‐old male carrying a homozygous mutation of SLC1A4 (p.Arg457Trp). Number of individual of the same gender for each spouse were indicated.

## 4 Conclusion and Results

This report presents a novel dual‐mutation case of homozygous *SLC1A4* and heterozygous *APOE4* variant in a patient with progressive microcephaly and spastic tetraplegia. Although the APOE ε4 allele was identified in this patient, the current case does not provide sufficient evidence to establish a causal or contributory role for this variant in the observed phenotype. The potential impact of APOE ε4 should therefore be interpreted with caution, and further family‐based and functional studies are needed to clarify any possible modifier effects. We suggest that a potential interplay between amino acid transport and lipid metabolism may have a synergistic detrimental impact on neurodevelopment. The homozygous *SLC1A4* mutation reported here is well‐documented in SPATCCM. The additional presence of a heterozygous *APOE* variant raises interesting questions about its potential role in exacerbating neurodevelopmental deficits, particularly in the context of an already disrupted metabolic pathway. Future research will be needed to address the effect of the combined variants on brain development, synaptic function, and myelination in an in vivo model. Such a model would provide insights into how amino acid and lipid metabolism cooperate and contribute to neurodevelopmental disorders. Understanding these mechanisms could pave the way for targeted therapeutic interventions aimed at restoring metabolic balance, improving neurological outcomes, and the patient's prognosis.

## 5 Discussion

Little is known about the critical metabolic changes of neurons undergoing development and differentiation in the case of SPATCCM. Since the first case reported in 2015 [2], several studies highlighted the role of SLC1A4/ASCT1 in the regulation of the neutral amino acid transport in the brain, with particular interest on L‐serine and D‐serine transport [19]. A knock‐in mouse model was generated for the most common mutation found in SPATCCM patients (p.E256K). The homozygous mice showed a marked microcephaly and thin corpus callosum, but normal motor coordination, learning and memory suggesting that the phenotypes observed in the human patients may be influenced by additional modifiers [20]. Odeh and colleagues recently characterized three mouse models of *Slc1a4* mutations: (i) a constitutive model with the complete deletion of *Slc1a4*; (ii) a knock‐in model carrying the human p.E256K mutation; (iii) a conditional knock‐out with selective deletion of *Slc1a4* in endothelial cells of the blood–brain barrier (BBB). This comparative study revealed a marked decrease in brain import of serine through the BBB, associated with microcephaly, synaptic impairment, dysregulation of sphingolipid synthesis and behavioral abnormalities, indicating a critical role of serine in the development and function of the central nervous system [21]. Accordingly, deficiency in the three serine‐biosynthetic enzymes (PHGDH, PSAT1 and PSPH) causes symptoms similar to ASCT1 deficiency, including congenital microcephaly, severe developmental delay and seizures [22]. L‐serine serves as a biosynthetic precursor to glycine and D‐serine, which are important for excitatory glutamatergic neurotransmission [23]. L‐serine modulates the mammalian target of rapamycin complex 1 (mTORC1), a crucial regulator of cellular metabolism, growth and differentiation [24]. In addition, L‐serine is required for the synthesis of sphingolipids and phosphatidylserine, which are essential for neuronal development [25], indicating a central bridge that links amino acid availability and lipid production and metabolism.

APOE is particularly abundant in the brain. Mostly secreted by astrocytes, it associates with lipoprotein particles and is mainly involved in lipid homeostasis, transport and metabolism. The APOE4 allele has been recognized as the strongest genetic risk factor for late‐onset Alzheimer's disease (AD) and has been linked to perturbations in neurovascular integrity, inflammation, oxidative stress, endosomal trafficking and calcium homeostasis [26]. In addition, APOE4 was associated with accumulation of cholesterol and sphingolipids in various brain areas in AD patients, but not in cognitively normal carriers [27]. Beyond its well‐known role in neurodegeneration, APOE4 has been suggested to have an impact on earlier developmental processes. Young adult APOE4 carriers display altered patterns of brain activity that can be detected before any sign of neurodegeneration [28]. Morphological brain abnormalities and impairments of intellectual performances have been detected in APOE4 carriers during childhood and adolescence [16, 17].

To our knowledge, this is the first case report of a SPATCCM patient with a dual‐variant scenario combining a homozygous *SLC1A4* mutation with heterozygous APOE4 allele variant. The hypothermia and liver injury were not reported in the previous *SLC1A4* published cases. Although the significant contribution of *SLC1A4* to microcephaly (through L‐serine transport deficiency) is well‐studied, it is still unknown whether the presence of an APOE4 allelic variant may exacerbate neuronal loss, synaptic impairment, and hypomyelination during an early period of life. It is worth noting that the release of APOE and lipids from microglial cells is L‐serine dependent [29], indicating a possible convergent molecular mechanism. Although the APOE ε4 allele was identified in this patient, the current findings do not establish a causal or modifying effect on the observed phenotype. The clinical manifestations are largely explained by the pathogenic SLC1A4 variants and are consistent with previously reported SPATCCM cases. Therefore, the APOE ε4 finding requires further investigation through family‐based and functional studies. A limitation of this report is the lack of trio whole‐exome sequencing, segregation analysis, and functional studies, which precludes assessment of whether the APOE ε4 allele has any modifying effect on the patient's phenotype.

## 6 Conclusions

This report presents a novel dual‐mutation case of homozygous *SLC1A4* and heterozygous *APOE4* variant in a patient with progressive microcephaly and spastic tetraplegia. Even though the pathogenic relevance of this putative double hit is still unknown, we suggest that a potential interplay between amino acid transport and lipid metabolism may have a synergistic detrimental impact on neurodevelopment. The homozygous *SLC1A4* mutation reported here is well‐documented in SPATCCM. The additional presence of a heterozygous *APOE* variant raises interesting questions about its potential role in exacerbating neurodevelopmental deficits, particularly in the context of an already disrupted metabolic pathway. Future research will be needed to address the effect of the combined variants on brain development, synaptic function, and myelination in an in vivo model. Such a model would provide insights into how amino acid and lipid metabolism cooperate and contribute to neurodevelopmental disorders. Understanding these mechanisms could pave the way for targeted therapeutic interventions aimed at restoring metabolic balance, improving neurological outcomes, and the patient's prognosis.

## Author Contributions

Mohammed Qaisiya: writing – original draft, investigation. Kenana Altell: writing – original draft, validation, Wasef Alhroub: writing – review and editing, conceptualization. Mahmoud Ramlawe: validation, visualization. Elisabetta Battocchio: writing – review and editing, resources. Fabrizia Guarnieri: writing – review and editing. Mu'taz Altamimi: visualization, supervision.

## Funding

The authors have nothing to report.

## Ethics Statement

This research did not require ethical approval, as the IRB committee does not mandate approval for reporting individual cases or case series.

## Consent

Written informed consent was obtained from the patient(s) for their anonymized information to be published in this article.

## Conflicts of Interest

The authors declare no conflicts of interest.

## Data Availability

The article contains all the data that support the study's findings and are readily accessible.
